# The Impact of Social Media Exposure and Interpersonal Discussion on Intention of COVID-19 Vaccination among Nurses

**DOI:** 10.3390/vaccines9101204

**Published:** 2021-10-19

**Authors:** Meiqi Xin, Sitong Luo, Rui She, Xi Chen, Liping Li, Lijuan Li, Xiaojun Chen, Joseph T. F. Lau

**Affiliations:** 1 Centre for Health Behaviours Research, School of Public Health and Primary Care, The Chinese University of Hong Kong, Hong Kong, China; meiqixin@link.cuhk.edu.hk (M.X.); sherryshe0319@link.cuhk.edu.hk (R.S.); 2 Vanke School of Public Health, Tsinghua University, Beijing 100083, China; sitongluo@mail.tsinghua.edu.cn; 3 Hunan Provincial Center for Disease Control and Prevention, Changsha 410007, China; chenxi161@sohu.com; 4 School of Public Health, Shantou University Medical College, Shantou 515041, China; lpli@stu.edu.cn; 5 School of Public Health, Dali University, Dali 671003, China; lelejuan@dali.edu.cn; 6 The First Affiliated Hospital of Shantou University Medical College, Shantou 515041, China; xjchen@stu.edu.cn

**Keywords:** social media, interpersonal discussion, vaccination intention, COVID-19, nurse

## Abstract

COVID-19 vaccine hesitancy among nurses is a global public health concern and it is imperative to understand associated factors. Information environment plays a critical role in shaping health behaviors, while few studies explored such effects in the context of COVID-19 vaccination. A cross-sectional survey was conducted among 1902 nurses in China. The study investigated the effects of social media exposure/interpersonal discussion on intention of COVID-19 vaccination and tested whether perceived effectiveness of COVID-19 vaccines (perceived vaccine efficacy, perceived duration of protection, and perceived effectiveness in preventing resurgences) mediated such associations. Results showed that about 68.0% and 56.5% of the participants had an intention of free and self-paid COVID-19 vaccinations, respectively. Frequent social media exposure and interpersonal discussion were positively associated with vaccination intentions. Perceived vaccine efficacy significantly mediated the effects of frequent social media exposure and interpersonal discussion, whereas perceived effectiveness in preventing resurgences suppressed the effects of frequent social media exposure. In conclusion, the prevalence of intention of COVID-19 vaccination was relatively low among Chinese nurses and health promotion is needed. Frequent social media exposure and interpersonal discussion potentially enhanced vaccination intentions via increased perceived vaccine efficacy. The findings can help inform the development of relevant health communication interventions.

## 1. Introduction

The COVID-19 pandemic has not been brought under control in most parts of the world, despite the widespread implementation of various non-pharmaceutical interventions, including personal protective measures and stringent restrictions (e.g., massive lockdown) [1]. As of 30 August 2021, there were nearly 216 million accumulated COVID-19 cases and 4.5 million deaths globally; the situation even became worse with a new peak emerging in the past two months [2]. Effective vaccination is deemed a promising strategy for fostering herd immunity in general populations [3]. COVID-19 vaccines have been developed at an unprecedented speed and many countries are implementing mass vaccination programs. During the last decade, vaccination hesitancy/resistance has become a global concern [4]. It is a major challenge for COVID-19 vaccination programs to reach a sufficient coverage required for achieving protection at the community level, as the expedition might have introduced concerns about the new vaccines’ performance and evaluation standards [5,6]. Some large-scale surveys found that around 72–74% of the general population accepted the COVID-19 vaccination [7,8]. As of 3 September 2021, only 40.1% of the world’s population has received at least one dose of a COVID-19 vaccine [9].

Healthcare workers are often prioritized in vaccination plans; their vaccination hesitancy is consequential as they are exposed to higher risk of COVID-19 infection [10]. Among them, nurses are particularly vulnerable to COVID-19 as they need to make frequent and close contact with patients [11,12]. Vaccinating nurses can also protect unvaccinated and immunocompromised patients from nosocomial infections [13,14]. Moreover, nurses are prospective promoters of COVID-19 vaccination, and their own vaccine hesitancy may diminish patients’ motivation to take up vaccination [15]. Despite the significance, nurses showed lower acceptance prevalence of COVID-19 vaccination (around 42–61%) than physicians (around 69–92%) [11,16,17]; one Hong Kong study reported that only 40% of Chinese nurses showed intention of COVID-19 vaccination [18]. Hence, promotion of COVID-19 vaccination among nurses and investigation of associated factors are greatly warranted.

According to the Social Learning Theory [19], the social environment plays a critical role in shaping health-related behaviors and intentions; such a learning process can take place through online and offline social interactions [20,21]. During the COVID-19 pandemic, some empirical studies have demonstrated that frequent social media exposure and/or interpersonal communication regarding COVID-19 were associated with adoption of preventive measures (e.g., face mask wearing and hand washing) [22,23,24,25]. However, only a relatively few studies have explored such relationships in the context of COVID-19 vaccination and yielded controversial findings. A Chinese study found that higher passive social media exposure, active social media interactions, and peer discussions were positively associated with intention of COVID-19 vaccination among university students [26]. Another study found that related social media exposure to information via social media showed a positive effect on intention of COVID-19 vaccination among the British population but not among Americans [27]. Some other studies found that social media users had lower intentions of COVID-19 vaccination than social media non-users and/or traditional media users [28,29,30].

Social media provide important interactive platforms for information seeking and sharing that can facilitate disaster preparedness [31]. Their functions are particularly crucial at times of public health emergencies [32]. Social media can be used to promote the public’s motivation to take up COVID-19 vaccination by disseminating timely information and updating information about the COVID-19 vaccination strategy (e.g., approvals and applications in various countries); in general, such information may reduce uncertainty and facilitate decision making about vaccination [33]. However, on the other side, social media messages often contain widespread misinformation, rumors, and anti-vaccine sentiments that would propagate vaccine hesitancy [34]. It is warranted to investigate how frequencies of exposure to related information via social media would affect intention of COVID-19 vaccination. 

Social media cannot replace direct interpersonal discussion, which takes place offline and involves one’s significant others, and may thus facilitate establishment of subjective norms [35,36]. When encountering uncertain situations and ambiguous information during a crisis, people tend to reduce their information gaps by seeking clarification, reassurance, and acquisition of firsthand experiences through their personal networks [37,38,39]. Mixed findings have been reported for the role of interpersonal discussion in improving behavioral responses to some previous emerging infectious diseases (e.g., the MERS outbreak in 2005 and the H1N1 influenza pandemic in 2009) [38,40,41]. To our knowledge, no studies have investigated the effects of social media exposure and interpersonal discussion regarding COVID-19 vaccination among nurses. 

Social media exposure and interpersonal discussion may be translated into intention of COVID-19 vaccination via modification of some key perceptions of the effectiveness of COVID-19 vaccines. Three dimensions are involved: (1) *perceived efficacy of COVID-19 vaccines* in protecting oneself which was found to increase intention of COVID-19 vaccination [42,43], (2) *perceived effectiveness of COVID-19 vaccination* in controlling the local epidemic which was found to increase intention of COVID-19 vaccination [44] and served as a major reason behind vaccination hesitancy among Chinese nurses [19], (3) *duration of COVID-19 vaccines’ protective effects* which was found to increase acceptability of human papillomavirus vaccination [45,46] but has not been explored in the context of COVID-19 vaccination. No studies have investigated whether these perceptions would mediate between social media exposure and interpersonal discussion, and COVID-19 vaccination intention. 

To address the research gaps, the present study aimed to first investigate the associations of free and self-paid COVID-19 vaccination with social media exposure and interpersonal discussion regarding COVID-19 vaccination among nurses, and to test whether such associations would be mediated through three dimensions of perceived effectiveness of COVID-19 vaccines (i.e., perceived vaccine efficacy, perceived duration of protection, and perceived effectiveness in preventing local resurgences). 

## 2. Materials and Methods

### 2.1. Study Procedures

During 19 October to 26 November 2020, a cross-sectional survey was conducted in five hospitals located in different regions of China (two in Hunan Province, two in Guangdong Province, and one in Yunnan Province). Eligible participants were full-time healthcare workers who had been employed by the participating hospitals since January 2020 (when COVID-19 first emerged) and were smartphone users. All eligible healthcare workers from the six major departments (medicine, surgeon, obstetrics and gynecology, pediatrics, infectious diseases, and emergency) of the participating hospitals were invited to join the survey. The hospital administrators assisted the research team in sending an invitation letter to the selected departments via the existing WeChat/QQ (the most popular social networking applications in China) groups where all healthcare workers joined as group members and communicated with each other about daily work.

Participants were first briefed about the study’s objectives, procedures, risks, and benefits (e.g., personal information will be collected as necessary and kept confidential; participants will not be subjected to physical harm during the survey) and informed that the participation was anonymous and voluntary, and they had the right to quit at any time. They were then asked to complete an online survey and were informed that return of the completed questionnaire implied informed consent. No incentives were provided. Ethical approval was obtained from the Ethics Committee of the corresponding author’s affiliated institution. A total of 3104 invitations were sent out and 2287 completed questionnaires were received (a response rate of 73.7%). With exclusion of 6 invalid responses and 17 cases that had taken up COVID-19 vaccination or made appointments, the effective sample size was 2264, including 362 doctors and 1902 nurses. The 1902 nurses were finally included in the analysis. 

### 2.2. Measures

#### 2.2.1. Socio-Demographics

Participants were asked to report their age, sex, marital status, education level, working department, and job title rank.

#### 2.2.2. Intention of COVID-19 Vaccination (Dependent Variables)

Two items were used to measure the intention of free and self-paid (RMB 600 or about USD 92) COVID-19 vaccination (having 80% efficacy and rare mild side effects) within six months since such vaccines would become accessible to the Chinese population, respectively. Participants were asked to rate the levels of intention on 5-point Likert scales (very unlikely, unlikely, neutral, likely, and very likely). The responses were dichotomized to represent whether having the intention of COVID-19 vaccination: 1 = yes (very likely/likely) and 0 = no (neutral/unlikely/very unlikely).

#### 2.2.3. Frequencies of Social Media Exposure about COVID-19 Vaccination (Independent Variables)

First, the “passive social media exposure” scale was formed by taking the average of two item responses (passively viewing related information on official and unofficial social media platforms); the Cronbach’s alpha was 0.95. Second, the “active social media exposure” scale comprised a single item of actively searching for information related to COVID-19 vaccination via social media. Participants were asked to rate the exposure frequencies in the past month on 5-point Likert scales (1 = very little to 5 = very much).

#### 2.2.4. Frequencies of Interpersonal Discussion about COVID-19 Vaccination (Independent Variables)

Two items were used to measure the frequencies of interpersonal discussion about COVID-19 vaccination in the past month with medical professionals and non-medical people, respectively, on 4-point Likert scales (1 = never to 4 = always).

#### 2.2.5. Perceptions of Effectiveness of COVID-19 Vaccines (Mediators)

Perceived vaccine efficacy was rated on a scale ranging from 0–100% (with 10% increments); the responses were recoded into 1 = 40% and below, 2 = 50%, 3 = 60%, 4 = 70%, 5 = 80%, and 6 = 90% and above. Perceived duration of protection was rated on an ordinal scale (<6 months, 6–12 months, 1–2 years, 2–5 years, >5 years, lifelong, and unknown); responses were dichotomized into 1 = 1 year and longer and 0 = else. Perceived effectiveness in preventing COVID-19 resurgences in China was rated on a 5-point Likert scale (1 = very low to 5 = very high).

### 2.3. Statistical Analysis

Descriptive analyses were presented. Univariate logistic regression analyses were conducted to test the associations of vaccination intentions with frequencies of social media exposure/interpersonal discussion and perceptions of effectiveness of COVID-19 vaccines; multivariate regression analyses were then conducted to test the above associations with adjustment for significant sociodemographic variables. Crude (COR) and adjusted (AOR) odds ratios, and their 95% confidence intervals (CI) were estimated. 

Structural equation modeling (SEM) was conducted to test the mediating effects of perceptions of effectiveness of COVID-19 vaccines on the associations between social media exposure/interpersonal discussion and intentions of free/self-paid vaccination. The two observed variables of “passive social media exposure” and “active social media exposure” were used as the manifest indicators of a latent variable “social media exposure”; the two observed variables of “discussion with medical professionals” and “discussion with non-medical people” were used as the manifest indicators of a latent variable “interpersonal discussion”. The other single items pertaining to perceptions of effectiveness of COVID-19 vaccines and vaccination intentions were directly included in the model as observed variables. 

With adjustment for significant sociodemographic variables, the path coefficients and 95% CIs were estimated with weight least square with mean and variance. The indirect effect and its 95% bias-corrected CI was estimated for each of the mediation pathways based on 5000 bootstrapped samples. Good model fit was represented by the Comparative Fit Index (CFI) > 0.95, the Tucker–Lewis Index (TLI) > 0.95, and the Root Mean Square of Approximation (RMSEA) < 0.06 [47]. A two-tailed *p* value < 0.05 indicated statistical significance. SEM analysis was conducted using Mplus 8.3 and the other analyses were conducted using SPSS Statistics 25.

## 3. Results

### 3.1. Sample Characteristics

Of the 1902 participants, 96.7% were females, 32.0% were married, 2.5% had a master’s degree, 56.5% worked in the departments of medicine and surgeon, and 45.1% had a rank title of middle and above. The mean (standard deviation (SD)) age of this sample was 31.72 (6.74). About 68.0% and 56.5% of the participants had the intention of free and self-paid COVID-19 vaccination, respectively. The mean score of frequencies of social media exposure/interpersonal discussion about COVID-19 vaccination ranged from 2.49 to 3.23. The mean score of perceived vaccine efficacy and perceived effectiveness in preventing resurgences ranged from 3.07 to 3.94; 48.3% perceived the protective effects lasting for 1 year or longer. Details are shown in Table 1.

### 3.2. Results of Logistic Regressions on Intentions of COVID-19 Vaccination

The univariate logistic regression analysis showed that compared with working in the department of medicine, working in the department of infectious diseases was positively associated with intention of free vaccination (COR = 1.72, 95% CI = 1.02 to 2.88, *p* = 0.04) while working in the department of obstetrics and gynecology was positively associated with intention of self-paid vaccination (COR = 2.00, 95% CI = 1.08 to 3.70, *p* = 0.03). Having a master’s degree was positively associated with intention of self-paid vaccination (COR = 1.90, 95% CI = 1.01 to 3.57, *p* = 0.04). The other sociodemographic variables were not associated with intention of free/self-paid vaccination. 

Adjusted for the significant background variables, the multivariate logistic regression analysis showed that frequencies of passive social media exposure (AOR = 1.31/1.40), frequencies of active social media exposure (AOR = 1.16/1.24), frequencies of discussion with medical professionals (AOR = 1.32/1.31), frequencies of discussion with non-medical people (AOR = 1.28/1.40), and the three types of perceptions of effectiveness of COVID-19 vaccines (AOR ranged from 1.12 to 1.44) were all positively associated with intention of free/self-paid vaccination. The association between perceived duration of protection and intention of self-paid vaccination showed a *p* value of 0.051. Details are shown in Table 2.

### 3.3. Results of Mediation Analysis

**Figure 1:**
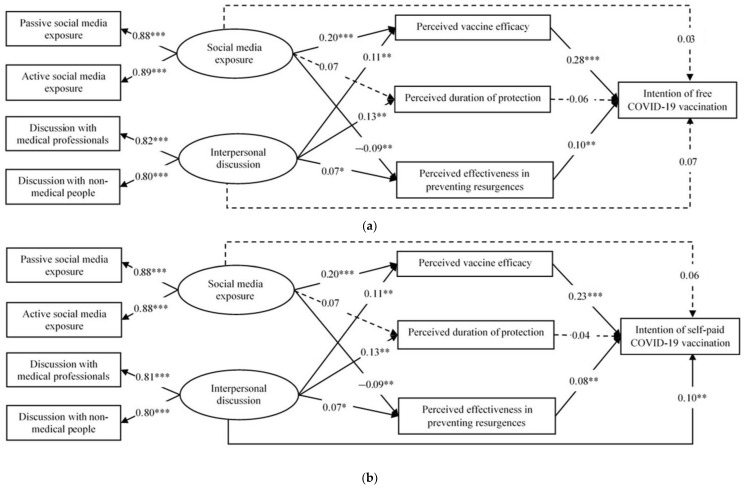
Structural equation modeling of the associations between frequencies of social media exposure/interpersonal discussion and intentions of COVID-19 vaccination mediated through perceptions of effectiveness of COVID-19 vaccines (*n* = 1902). * *p* < 0.05; ** *p* < 0.01; *** *p* < 0.001. (**a**) Outcome: Intention of free COVID-19 vaccination; (**b**) Outcome: Intention of self-paid COVID-19 vaccination.

The SEM models were well supported with good model fit for both outcomes (intention of free vaccination: CFI = 0.968, TLI = 0.957, and RMSEA = 0.024; intention of self-paid vaccination: CFI = 0.968, TLI = 0.957, and RMSEA = 0.023). The factor loadings of the two latent variables were highly significant (social media exposure: β = 0.88 to 0.89; interpersonal discussion: β = 0.80 to 0.82; *p* < 0.001). The standardized coefficients of all the structural paths were shown in Figure 1. The total effects of social media exposure on intention of free and self-paid vaccination were 0.080 (*p* = 0.03) and 0.101 (*p* = 0.004), respectively; those of interpersonal discussion were 0.115 (*p* = 0.003) and 0.132 (*p* < 0.001), respectively. 

Regarding the indirect effects of the mediators on intention of free COVID-19 vaccination (Table 3), (1) perceived vaccine efficacy significantly mediated both the associations between social media exposure (β = 0.056, *p* < 0.001)/interpersonal discussion (β = 0.047, *p* < 0.001) and vaccination intention; (2) perceived effectiveness in preventing resurgence significantly suppressed the association between social media exposure and vaccination intention (β = −0.009, *p* < 0.001) but did not significantly mediate the association between interpersonal discussion and vaccination intention (β = 0.007, *p* = 0.07); (3) perceived duration of protection did not show any significant mediating/suppressing effect. The pattern of the indirect effects on intention of self-paid vaccination was the same as the aforementioned model involving free vaccination. In addition, interpersonal discussion showed a significant direct effect on intention of self-paid vaccination intention (β = 0.097, *p* = 0.007) but not intention of free vaccination (β = 0.071, *p* = 0.07); social media exposure had no direct effects on both intention outcomes.

## 4. Discussion

This study suggested that only about 68% and 57% of the nurses in China intended to take up free and self-paid COVID-19 vaccinations with 80% efficacy and rare side effects, respectively. Previous studies conducted in various countries presented comparable acceptance rates of 42–61% for COVID-19 vaccination among nurses [11,17,18]. The prevalence is less than ideal, given the risk of nosocomial infections. Prompt health promotion is needed.

The key finding is that frequent social media exposure regarding COVID-19 vaccination was associated with increased vaccination intentions. During the past few months, the Chinese government has launched rumor-refuting websites, rectification campaigns, and punitive regulations against the spread of misinformation related to COVID-19 via social media platforms [48,49]. The information environment is thus more likely to be pro-vaccine in nature. Additionally, nurses may be inclined to access authorized and professional information sources on social media, which enables them to obtain accurate and scientific information about COVID-19 vaccination. Likewise, frequent interpersonal discussion with both medical professionals and non-medical people about COVID-19 vaccination were positively associated with intention of COVID-19 vaccination. Interestingly, frequent discussion with medical professionals did not exert particularly larger effects when compared to frequent discussion with laymen. Thus, the public’s views may be as important as professional knowledge in influencing COVID-19 vaccination intentions.

The SEM analysis further suggested that frequent social media exposure and interpersonal discussion had independent effects on intention of free/self-paid COVID-19 vaccination, while interpersonal discussion appeared to have a larger effect size after controlling for each other. Compared with information exposure via social media, interpersonal discussion often involved more narratives of personal experiences given by socially proximal others, which may increase perceived self-relevance and reduce counterarguing against the advocacy [50,51]. In addition, interpersonal discussion can reinforce social norms and facilitate systematic information processing, and consequently enhance the intention of adopting preventive behaviors [38,40]. The implication is that both online social marketing and structured interpersonal discussion in offline settings (e.g., hospital seminars) are warranted to promote COVID-19 vaccination among nurses. Pilot randomized controlled studies may test the efficacy of interventions using both online and offline popular opinion leaders [52] to disseminate pro-vaccine messages among nurses.

This study corroborates previous findings that perceptions of effectiveness of COVID-19 vaccines were positively associated with vaccination intentions. It elaborates on two types of personal concerns (vaccine efficacy and duration of protection) and the more “collective” and “altruistic” community-oriented concern (effectiveness in preventing resurgences in the community). Interventions via social media and interpersonal discussion shall educate about different aspects of vaccine effectiveness. Phase III clinical trials can inform about personal protectiveness, through which inference about community protection can also be made. There is, however, little information about duration of immunity; such data need to be updated as soon and as far as possible.

It is important to understand the mechanisms of how frequent social media exposure and interpersonal discussion regarding COVID-19 vaccination might affect vaccination intentions. The SEM found similar results for free and self-paid vaccination. First, although frequent interpersonal discussion was positively associated with perceived duration of protection, this perception variable was not a mediator as its associations with vaccination intentions were non-significant, possibly because of its correlations with the other two types of perceived effectiveness (i.e., efficacy and duration of protection). Second, only perceived vaccine efficacy but not perceived effectiveness in preventing resurgences significantly mediated the associations between frequent interpersonal discussion and vaccination intentions. Third, perceived effectiveness in preventing resurgences even suppressed the association between frequent social media exposure and vaccination intentions as it was negatively associated with social media exposure. The suppressing effect is unexpected and indicates that social media may not be consistently supportive of different aspects of COVID-19 vaccination. It is plausible that there are some uncertainties about the practical effectiveness of the vaccination strategy in protecting communities, which may depend more on other contextual factors such as a sufficient population coverage [53]. The contention needs to be confirmed by investigating not only exposure frequencies but also contents of related social media messages.

In general, the SEM results support the social learning theory that the social information environment can influence individuals’ perceptions of a recommended behavior and enhance motivations to enact the behavior [19]. It also suggests that relevant intervention messages may focus on the efficacy of COVID-19 vaccines in self-protection. Furthermore, although perceived vaccine efficacy was found to fully mediate the effect of frequent social media exposure on vaccination intentions, the direct effect of interpersonal discussion on intention of self-paid vaccination remained significant, which indicates the existence of other potential mediators, such as positive emotional reactions to vaccination [54], need for adequate information [55], and supportive social norms [56].

Our findings have implications for promotion of COVID-19 vaccination among nurses across different contexts. Healthcare workers are prioritized for COVID-19 vaccination in China [57] and some other countries as well [58]. Despite intensive financial and policy support to boost COVID-19 vaccination (e.g., securing sufficient supplies to all the healthcare workers [59]; imposing vaccine requirements and related working restrictions [60]), a sizable proportion of healthcare workers (including nurses) are hesitant to receive the vaccination [58]. The results of this study potentially apply to the countries with high availability of COVID-19 vaccines to nurses, and to both the cases where individuals intend to receive a free vaccine and purchase a non-funded vaccine. The similar mediation patterns observed for the two intention outcomes suggest that social media exposure and interpersonal discussion may consistently exert their effects on nurses’ intention of free and self-paid vaccination through perceived effectiveness of the COVID-19 vaccines. After all, the existing level of vaccine hesitancy within social networks and the nature of relevant social media content can vary across countries and regions [58,61]. It is hence warranted to examine the effect and mechanism of social media exposure and interpersonal discussion among other nursing populations.

The study has some limitations. First, the casual relationships between the studied variables cannot be concluded by the cross-sectional study design. Second, the specific contents of social media exposure and interpersonal discussion regarding COVID-19 vaccination were not measured, hence, it is unable to explore the influences of information valence and quality (e.g., reliability and accuracy). Third, the online self-administered survey might cause reporting bias. Fourth, some single items were used to assess the perceptions of vaccines. Finally, the generalizability of our findings to other countries should be cautiously made.

## 5. Conclusions

The present study indicates that Chinese nurses have relatively low intentions to take up free and self-paid COVID-19 vaccination, even if the vaccines exhibit 80% efficacy and rare side effects. Both frequent social media exposure and interpersonal discussion had a significant association with higher vaccination intentions. The mediation analyses further demonstrated the differential mediating roles of three aspects of perceptions of effectiveness of COVID-19 vaccines. Perceived vaccine efficacy mediated the effect of frequent social media exposure/interpersonal discussion from frequent social media exposure/interpersonal discussion whereas perceived effectiveness in preventing resurgences suppressed the effects of frequent social media exposure. Perceived duration of protection did not have a significant mediation effect. The findings highlight the importance of developing effective communication interventions for vaccination promotion among nurses. The study also informs the future investigation of the hypothesized relationships in other contexts with different vaccine acceptance levels.

## Figures and Tables

**Table 1:** Sample characteristics (*n* = 1902).

Variables	*n* (%)/Mean ± SD
**Sociodemographic variables**	
Sex	
Male	62 (3.3)
Female	1840 (96.7)
Age (years old)	31.72 ± 6.74
Marital status	
Married	608 (32.0)
Single/divorced/widowed/others	1294 (68.0)
Education level	
Bachelor’s degree and below	1854 (97.5)
Master’s degree	48 (2.5)
Department	
Medicine	560 (29.4)
Surgeon	515 (27.1)
Obstetrics and gynecology	55 (2.9)
Pediatrics	210 (11.0)
Infectious diseases	93 (4.9)
Emergency	154 (8.1)
Others	315 (16.6)
Job title rank	
Primary	1045 (54.9)
Middle	726 (38.2)
Vice-senior/Senior	86 (4.5)
Senior	5 (0.3)
Others	40 (2.1)
**Intention of COVID-19 vaccination**	
Intention of free COVID-19 vaccination	
No	608 (32.0)
Yes	1294 (68.0)
Intention of self-paid COVID-19 vaccination	
No	828 (43.5)
Yes	1074 (56.5)
**Frequencies of social media exposure about COVID-19 vaccination**	
Passive social media exposure	3.23 ± 0.87
Active social media exposure	3.06 ± 0.93
**Frequencies of interpersonal discussion about COVID-19 vaccination**	
Discussion with medical professionals	2.65 ± 0.77
Discussion with non-medical people	2.49 ± 0.79
**Perceptions of effectiveness of COVID-19 vaccines**	
Perceived vaccine efficacy	3.94 ± 1.43
Perceived duration of protection	
≥1 year	919 (48.3)
Else	983 (51.7)
Perceived effectiveness in preventing resurgences	3.07 ± 1.09

**Table 2:** Logistic regressions on intentions of COVID-19 vaccination (*n* = 1902).

Variables	Intention of Free COVID-19 Vaccination		Intention of Self-Paid COVID-19 Vaccination	
	Crude Odds Ratio (95% CI)	Adjusted Odds Ratio (95% CI)	Crude Odds Ratio (95% CI)	Adjusted Odds Ratio (95% CI)
**Frequencies of social media exposure about COVID-19 vaccination**				
Passive social media exposure	1.30 (1.16, 1.46) ***	1.31 (1.17, 1.46) ***	1.40 (1.26, 1.56) ***	1.40 (1.26, 1.56) ***
(1) Passive exposure via official platforms	1.28 (1.15, 1.43) ***	1.29 (1.16, 1.44) ***	1.40 (1.26, 1.56) ***	1.40 (1.26, 1.56) ***
(2) Passive exposure via unofficial platforms	1.29 (1.16, 1.44) ***	1.29 (1.16, 1.44)	1.36 (1.22, 1.50) ***	1.36 (1.22, 1.51) ***
Active social media exposure	1.15 (1.04, 1.28) **	1.16 (1.04, 1.28) **	1.24 (1.12, 1.36) ***	1.24 (1.12, 1.37) ***
**Frequencies of interpersonal discussion about COVID-19 vaccination**				
Discussion with medical professionals	1.32 (1.16, 1.50) ***	1.32 (1.16, 1.50) ***	1.30 (1.15, 1.46) ***	1.31 (1.16, 1.48) ***
Discussion with non-medical people	1.28 (1.13, 1.44) ***	1.28 (1.13, 1.44) ***	1.39 (1.24, 1.57) ***	1.40 (1.25, 1.58) ***
**Perceptions of effectiveness of COVID-19 vaccines**				
Perceived vaccine efficacy	1.43 (1.33, 1.54) ***	1.44 (1.34, 1.55) ***	1.37 (1.28, 1.46) ***	1.37 (1.28, 1.47) ***
Perceived duration of protection				
≥1 year	1.22 (1.01, 1.48) *	1.25 (1.03, 1.52) *	1.18 (0.98, 1.41) †	1.20 (1.00, 1.44) †
Else	Ref	Ref	Ref	Ref
Perceived effectiveness in preventing resurgences	1.15 (1.05, 1.26) **	1.15 (1.06, 1.26) **	1.12 (1.03, 1.21) **	1.12 (1.03, 1.22) **

Note: Adjusted odds ratios were calculated for the outcome of intention of free COVID-19 vaccination with adjustment for department and were calculated for the outcome of intention of self-paid COVID-19 vaccination with adjustment for department and education level. † *p* < 0.1; * *p* < 0.05; ** *p* < 0.01; *** *p* < 0.001.

**Table 3:** The effects of the pathways from frequencies of social media exposure/interpersonal discussion to intentions of COVID-19 vaccination based on 5000 bootstrapped samples.

Pathways	Intention of Free COVID-19 Vaccination		Intention of Self-Paid COVID-19 Vaccination	
	β	*p* Value	β	*p* Value
**Independent variable (IV): social media exposure**				
1. Total effect: IV → outcome	0.080	0.03 *	0.101	0.004 **
2. Direct effect: IV → outcome	0.029	0.41	0.059	0.09
3. Indirect effect:				
(1) IV → perceived vaccine efficacy → outcome	0.056	<0.001 ***	0.047	<0.001 ***
(2) IV → perceived duration of protection → outcome	0.004	0.31	0.002	0.45
(3) IV → perceived effectiveness in preventing resurgences → outcome	−0.009	0.03 *	−0.007	0.04 *
**IV: interpersonal discussion**				
1. Total effect: IV → outcome	0.115	0.003 **	0.132	<0.001 ***
2. Direct effect: IV → outcome	0.071	0.07	0.097	0.007 **
3. Indirect effect:				
(1) IV → perceived vaccine efficacy → outcome	0.030	0.004 **	0.025	0.005 **
(2) IV → perceived duration of protection → outcome	0.007	0.20	0.005	0.38
(3) IV → perceived effectiveness in preventing resurgences → outcome	0.007	0.07	0.005	0.10

* *p* < 0.05; ** *p* < 0.01; *** *p* < 0.001.

## Data Availability

The data presented in this study are available on request from the corresponding author. The data are not publicly available due to privacy and ethical concerns.
